# Sensor-Model Matching for Controlled Comparison of Bayesian and Belief-Function Occupancy Grid Fusion

**DOI:** 10.3390/s26134266

**Published:** 2026-07-04

**Authors:** Tatiana Berlenko, Kirill Krinkin

**Affiliations:** 1 School of Computer Science and Engineering, Constructor University, Campus Ring 1, 28759 Bremen, Germany; tberlenko@constructor.university; 2 JetBrains, Paphos 8046, Cyprus

**Keywords:** occupancy grid mapping, Dempster–Shafer theory, Bayesian fusion, belief functions, pignistic transform, sensor model equivalence

## Abstract

Comparisons of Bayesian log-odds and Dempster’s combination rule for occupancy grid mapping typically parameterize the two sensor models independently, so that observed performance differences confound the fusion rule with the sensor parameterization. We develop a pignistic-transform-based matching methodology that derives belief function masses producing identical per-observation decision probabilities, isolating the accumulation rule as the sole variable. We show that the confound is large: in multi-robot experiments under two noise conditions, applying the match reversed boundary sharpness from a +6% to +14% advantage for belief functions to a −17% to −22% deficit favoring Bayesian log-odds—a 23 to 36 percentage-point reversal, consistent across both conditions—motivating per-observation matching as the basis for controlled comparison. Under BetP-matched comparison in single-agent simulation (15 independent runs) and on two real indoor lidar datasets (Intel Research Lab, Freiburg Building 079), the two frameworks produce practically equivalent maps on the reported point-probability metrics (cell accuracy, boundary sharpness, Brier score), with a small directional advantage for Bayesian log-odds (absolute differences 0.001–0.022 on [0, 1] scales). Under normalized plausibility ($P_{\mathrm{Pl}}$) matching, the direction reverses for boundary sharpness and Brier score, indicating that the ranking depends on the probability transform used for matching, not solely on the fusion rule. All evaluation is restricted to point-probability metrics on 2D binary grids with Dempster’s and Yager’s rules. The interval-valued representation [Bel(A),Pl(A)] unique to belief functions is not assessed. The matching methodology is applicable to other Bayesian/belief function comparisons.

## 1. Introduction

Occupancy grid mapping is a standard spatial representation in mobile robotics, in which each cell stores an occupancy probability updated incrementally from sensor observations. The Bayesian log-odds formulation [1,2] is the default update mechanism in widely deployed SLAM systems [3,4,5]. An alternative formulation based on Dempster–Shafer belief functions [6,7] and the Transferable Belief Model [8,9] represents each cell as a mass triplet and has been applied with reported advantages in dynamic object detection [10] and evidential SLAM [11]. Despite both approaches being well-established, no prior comparison has isolated the effect of the fusion rule from the sensor model parameterization.

Prior studies assign log-odds increments and mass values independently, so that differences in per-observation aggressiveness can mask or reverse the accumulation rule effect. We show that this confound is large enough to reverse the direction of a fusion rule comparison entirely (Section 3.1).

This paper introduces a methodology that controls for the sensor-model confound and characterizes what remains once the sensor model effect is fixed. Our contributions are:

1. **Pignistic matching methodology.** A pignistic-transform-based procedure that matches per-observation decision probabilities across frameworks, enabling controlled comparison independent of sensor parameterization (Section 3.2).
2. **Confound demonstration.** Independent parameterization from community defaults produces a moderate mismatch (BetP(O)=0.825 vs. $\sigma (l_{\mathrm{occ}})=0.881$) that reversed boundary sharpness by 23–36 percentage points across two noise conditions in our experiments (Section 3.1).
3. **BetP-matched empirical comparison.** Under matched comparison in single-agent simulation and on two real indoor lidar datasets, the two frameworks produce practically equivalent maps with a small directional advantage for Bayesian log-odds (absolute differences 0.001–0.022 on [0,1] scales; Section 5).
4. **Transform dependence.** Under normalized plausibility ($P_{\mathrm{Pl}}$) matching, the direction reverses for boundary sharpness and Brier score (Section 5.3).

Scope limitations are discussed in Section 6.

Section 2 reviews related work. Section 3 develops the matching methodology. Section 4 describes the experimental setup. Section 5 presents simulation and real-data results. Section 6 discusses implications and limitations. Section 7 concludes.

## 2. Related Work

This section reviews the Bayesian and belief function approaches to occupancy grid mapping and identifies a methodological gap in prior comparison studies.

### 2.1. Bayesian Occupancy Grid Mapping

Elfes [1] introduced the occupancy grid as a spatial representation for mobile robot perception, in which each cell stores an independent occupancy probability updated from sensor observations via Bayes’ rule. Thrun et al. [2] systematized the approach within the broader framework of probabilistic robotics, introducing the log-odds representation L=log(p/(1−p)) that converts multiplicative Bayesian updates into efficient additive operations. This formulation, combined with clamping of accumulated log-odds to a symmetric bound $\left|L\right|\leq L_{\max}$ for numerical stability, has become the standard implementation in the robotics community.

The Bayesian log-odds approach is the default in widely deployed open-source SLAM systems, including GMapping [3] (Rao–Blackwellized particle filter SLAM), Google Cartographer [4] (submap-based graph SLAM), and SLAM Toolbox [5] (the Robot Operating System (ROS) 2 navigation stack default). Its popularity rests on simplicity (one floating-point value per cell), computational efficiency (one addition per update), and the well-understood Bayesian interpretation of the resulting probabilities. The formal treatment of Bayesian occupancy grid fusion is given in Section 3.

### 2.2. Belief Function Occupancy Grid Mapping

An alternative line of work applies Dempster–Shafer (DS) belief function theory [6,7,12] to occupancy grid mapping, replacing the single occupancy probability with a basic belief assignment (BBA) that explicitly represents ignorance. Within the Transferable Belief Model (TBM) [8], each cell stores a mass triplet $\left(m_{O},\;m_{F},\;m_{OF}\right)$ where $m_{OF}$ quantifies the degree of epistemic ignorance—mass not yet committed to either hypothesis. Decisions are made via the pignistic transform [9]: under the closed-world assumption (m(∅)=0), the pignistic probability of occupancy reduces to $\mathrm{BetP}\left(O\right)=m_{O}+m_{OF}/2$, mapping belief functions to probabilities for action selection. Cuzzolin [13] provides a modern geometric treatment of the relationship between belief functions and probability, situating the pignistic transform within a broader family of probability transforms on the belief space.

Moras et al. [10,14] developed *credibilist occupancy grids* for vehicle perception in dynamic traffic environments, proposing the use of conflict mass *K* between successive observations as a metric for detecting moving objects. Their approach demonstrated that cells affected by dynamic obstacles produce elevated conflict under Dempster’s combination rule, providing a mechanism for dynamic detection that has no direct analogue in standard Bayesian grids.

Huletski et al. [11] presented vinySLAM, an indoor SLAM system that replaces the single-probability cell model of tinySLAM with TBM-based mass triplets, targeting resource-constrained platforms such as the Raspberry Pi. The system demonstrated accuracy improvements over tinySLAM on indoor benchmarks. However, vinySLAM simultaneously modified both the occupancy cell model (from Bayesian to TBM) and the scan matching cost function (adding angle-histogram-based weighting). No ablation study has been published that isolates the contribution of the TBM cell model from the improved scan matching, leaving the source of the accuracy improvement ambiguous (disclosure: the second author of the present work is a co-author of [11]).

Nuss et al. [15] proposed a random finite set (RFS) approach extending evidential occupancy modelling to dynamic multi-object scenarios. Ben Ayed et al. [16] provide a recent survey of evidential occupancy grid methods.

Reported advantages of the belief function approach include dynamic object detection via conflict analysis [10], richer uncertainty representation through the ignorance mass $m_{OF}$ (distinguishing unobserved from conflicting cells [8]), and interval-valued belief–plausibility bounds for conservative decision-making. These advantages are theoretically well-motivated. The question addressed here is whether they translate to measurable performance differences on point-probability metrics under controlled comparison with matched sensor models.

#### Alternative Combination Rules

Dempster’s rule is one of several operators for combining belief functions. Its conflict normalization by 1/(1−K) has been discussed since Zadeh’s [17] observation that it can produce counterintuitive results under high conflict. Alternatives include Yager’s rule [18] (conflict assigned to ignorance), Dubois–Prade [19] (disjunctive redistribution), Denœux’s cautious rule [20], and PCR [21]. Our analysis tests Dempster’s and Yager’s rules, the two most common in the evidential occupancy grid literature; generalization to other rules is future work.

### 2.3. Comparison Studies and the Sensor Model Gap

Despite the growing body of work on belief function occupancy grids, controlled comparisons between the two frameworks remain scarce, and those that exist share a common methodological limitation: sensor model parameters are chosen independently for each framework. In the credibilist grid literature [10,14], Bayesian log-odds and belief function masses are set separately; in vinySLAM [11], the TBM cell model and scan matching cost function were changed simultaneously without ablation; the RFS-based approach [15] uses observation models specific to the RFS framework. In all cases, the absence of a formal matching criterion means that observed performance differences reflect an unknown mixture of sensor model and fusion rule effects (detailed critique in Supplementary Material).

#### The Methodological Gap

To our knowledge, no prior comparison of Bayesian and belief function occupancy grid fusion has applied per-observation sensor model matching via the pignistic transform. This matching—requiring that the pignistic probability BetP(O) equal the Bayesian posterior σ(l) for each individual sensor observation—ensures that any aggregate performance difference is attributable to the multi-observation accumulation rule (additive log-odds with clamping versus Dempster’s combination with conflict normalization), not to the single-observation sensor model. This methodology is developed in Section 3 and constitutes one of the main contributions of the present work.

We emphasize that the absence of per-observation matching in prior work is a previously unrecognized methodological confound, not an error by prior authors. The belief function and Bayesian communities have traditionally parameterized their sensor models according to framework-specific conventions, and there has been no established procedure for cross-framework matching. The pignistic transform provides a natural and principled bridge, but its application to sensor model matching for controlled comparisons appears to be novel. Customizable frameworks for evidential occupancy grids have been proposed [22], but per-observation matching between Bayesian and belief function sensor models has not been addressed.

## 3. Matching Methodology

A meaningful comparison of Bayesian and belief function fusion requires that any observed performance difference be attributable to the fusion rule itself, not to differences in per-observation sensor model parameterization. This section develops a matching methodology based on the pignistic transform [9] that ensures per-observation equivalence at the decision-probability level. The matching equates pignistic probabilities, not the belief function representations themselves; it is therefore BetP-specific. Alternative transforms (normalized plausibility, maximum entropy) yield different matched masses and a different comparison (Section 5.3).

The methodology rests on a bijection φ:R→M, where M denotes the set of consonant basic belief assignments on the binary frame {O,F}, mapping scalar Bayesian log-odds to mass triplets. The explicit form of φ and the proof of bijectivity are given as Theorem 1 in Section 3.2. This bijection holds at the single-observation level; under repeated observations, the two accumulation mechanisms diverge.

### 3.1. The Sensor Model Equivalence Problem

In initial multi-robot experiments (three robots, 15 independent runs under each of two noise conditions), belief function fusion achieved a +6% to +14% boundary sharpness advantage over Bayesian fusion (+14% under the dynamic-baseline condition, +6% under elevated sensor noise). After applying the pignistic transform matching procedure described below—which constrains the belief function masses to produce identical per-observation decision probabilities—the same experiments yielded a −17% to −22% boundary sharpness *deficit* for belief function fusion (−22% under the dynamic-baseline condition, −17% under elevated sensor noise), with 15/15 directional consistency favoring the Bayesian arm in each condition (per-condition before/after values in Supplementary Material, Section S5). This reversal—a 23 to 36 percentage-point swing—was caused entirely by the sensor model parameterization, not by any change to the fusion algorithms themselves. This motivates a formal matching criterion: the per-observation update must produce *identical decision probabilities* under both frameworks before any multi-observation accumulation occurs.

### 3.2. Pignistic Transform Matching

We derive matched belief function masses from Bayesian log-odds parameters using the pignistic probability transform [9]. Given a basic belief assignment *m* on the binary frame Θ={O,F}, the pignistic probability of occupancy is

(1) $$\mathrm{BetP}\left(O\right)=m_{O}+\frac{m_{OF}}{2}$$

(see Section 2 for background).

In the Bayesian framework, a single observation with log-odds *l* produces the occupancy probability p=σ(l), where $\sigma \left(l\right)=1/\left(1+e^{-l}\right)$ is the logistic sigmoid. The matched masses derived below are the constructive application of the bijection φ (see above): given *l*, the unique consonant BBA satisfying BetP(O)=σ(l) is precisely φ(l). The matching condition requires

(2) BetP(O)=σ(l),

for each observation type (occupied hit or free traversal).

Setting p=σ(l) and requiring that $m_{O}+m_{F}+m_{OF}=1$ with all masses non-negative, the unique solution is:

(3) $$\begin{array}{cc} m_{O} & =\max(0,\;2p-1), \end{array}$$

(4) $$\begin{array}{cc} m_{F} & =\max(0,\;1-2p), \end{array}$$

(5) $$\begin{array}{cc} m_{OF} & =1-|2p-1|. \end{array}$$

**Theorem 1** 
(Single-observation pignistic equivalence)**.** *The map φ defined by* (3)–(5) *is a bijection between scalar log-odds and consonant BBAs on* {O,F} *that satisfies* BetP(O)=σ(l) *for every* l∈R*.*

**Proof.**  *(i) Validity.* For l≥0: $\sigma \left(l\right)\in \left[\frac{1}{2},1\right)$, so $m_{O}=2\sigma \left(l\right)-1\geq 0$, $m_{F}=0$, $m_{OF}=2\left(1-\sigma \left(l\right)\right)>0$. Sum: (2σ(l)−1)+0+2(1−σ(l))=1. The case l<0 is symmetric.

*(ii) BetP recovery.* For l≥0: $\mathrm{BetP}\left(O\right)=m_{O}+m_{OF}/2=\left(2\sigma \left(l\right)-1\right)+\left(1-\sigma \left(l\right)\right)=\sigma \left(l\right)$. For l<0: BetP(O)=0+σ(l)=σ(l).

*(iii) Bijectivity.* The inverse is $\varphi^{-1}\left(m\right)=\log\frac{\mathrm{BetP}(O)}{1-\mathrm{BetP}(O)}$. Since σ is bijective R→(0,1) and BetP(O)∈(0,1) for any consonant BBA with $m_{OF}>0$, the composition is bijective. □

Further properties of the matching—closed-form accumulation divergence and monotonic decay of $m_{OF}$—are proved in the Supplementary Material, Sections S2.2 and S2.3.

#### 3.2.1. Choice of Probability Transform

We adopt BetP because it is the standard decision-theoretic choice within the TBM [9] and the most widely used transform in the evidential occupancy grid literature [10,11]. The matching is BetP-specific: the normalized plausibility transform $P_{\mathrm{Pl}}\left(O\right)=\mathrm{Pl}\left(O\right)/\left(\mathrm{Pl}\left(O\right)+\mathrm{Pl}\left(F\right)\right)$ yields systematically lower ignorance mass per observation than BetP (approximately 44% less at the default sensor parameters, $l_{\mathrm{occ}}=2.0$), producing sharper DS updates. Under multi-observation accumulation, the combined BBA becomes non-consonant at boundary cells (where both $m_{O}>0$ and $m_{F}>0$), and the two transforms diverge further. Our evaluation is therefore BetP-specific throughout; the quantitative impact of the transform choice is assessed in the $P_{\mathrm{Pl}}$ sensitivity analysis of Section 5.3.

#### 3.2.2. Consonant Mass Functions

The pignistic-derived masses are consonant ($m_{F}=0$ for occupied, $m_{O}=0$ for free observations), reflecting the physical constraint of single-source inverse sensor models: a lidar beam returns evidence for *either* occupancy or free space, never both simultaneously. All prior DS occupancy grid work cited here [10,11,14] uses consonant BBAs for the same reason. Non-consonant BBAs from multi-source fusion fall outside this scope.

### 3.3. Per-Observation Locality

A critical property of the pignistic matching is that it holds *per-observation:* at each specific sensor distance *d* and angle of incidence α, the matching condition (2) is satisfied independently.

In the single-agent simulation experiment (Section 4.1), the sensor model is non-homogeneous: log-odds values decay exponentially with distance and angle of incidence,

(6) $$l\left(d,\alpha \right)=l_{\mathrm{obs}}\cdot \exp\left(-\lambda_{d}\;d\right)\cdot \exp\left(-\lambda_{\alpha }\;\alpha \right),$$

where $l_{\mathrm{obs}}\in \left\{l_{\mathrm{occ}},l_{\mathrm{free}}\right\}$ and $\left(\lambda_{d},\lambda_{\alpha }\right)=\left(0.1,0.5\right)$ are the distance and angle decay rates respectively. Because the pignistic-derived masses are computed from p=σ(l(d,α)) at each (d,α) pair individually, the matching holds at every observation regardless of the spatial variation in sensor confidence. The single-observation bijection therefore applies locally to each per-observation update, even though the global sensor model is non-homogeneous.

This per-observation locality is the key property that enables fair comparison under realistic sensor models. It means that any aggregate performance difference between the two frameworks within a single experimental setup is attributable to the multi-observation *accumulation rule*—Bayesian log-odds addition with clamping versus Dempster’s combination with conflict normalization—not to the single-observation updates.

The multi-robot simulation, mechanism test, and real-data (scan-split) experiments use constant sensor parameters ($l_{\mathrm{occ}}=2.0$, $l_{\mathrm{free}}=-0.5$) with pignistic-matched constant masses. The per-observation locality argument still holds—it simply reduces to a single matching point rather than a continuous function of (d,α). The non-homogeneous model is exercised only in the single-agent experiment to demonstrate that the methodology extends to spatially varying sensor confidence; the real-data sensor model is not calibrated to the SICK LMS200 specifically. The sensor parameter sensitivity analysis (Supplementary Material, Section S7) tests four parameterizations spanning the typical indoor lidar range (weak, default, strong, symmetric); the Bayesian advantage is directionally consistent across all four, with the smallest separation under the symmetric configuration ($|l_{\mathrm{occ}}\left|=\right|l_{\mathrm{free}}|$). Extreme values outside this range (e.g., $l_{\mathrm{occ}}>5$ or near-zero evidence) were not tested and could exhibit different behavior. The real-data experiments use only the default parameterization; real-data sensitivity to sensor parameters remains untested.

### 3.4. Accumulation Divergence

Although per-observation updates are identical by construction, the two frameworks diverge under repeated observations. The Bayesian arm clamps at $\left|L\right|\leq L_{\max}$ (saturation at N=5 for $L_{\max}=10$, $l_{\mathrm{occ}}=2.0$); the DS arm converges asymptotically to BetP→1.0 with no ceiling. At boundary cells with conflicting observations, Dempster’s 1/(1−K) normalization converges more slowly than additive log-odds (e.g., BetP=0.9973 vs. p=0.9994 after 5 occupied and 5 free observations). Detailed convergence analysis is in Supplementary Material, Section S3.

## 4. Experimental Setup

We evaluate the two fusion frameworks in single-agent simulation and on two real indoor lidar datasets. A multi-robot simulation (3 robots with PGO alignment, two noise conditions, 15 runs each) is presented in the Supplementary Material, Section S5. The sensor models differ between experiment types by design. The single-agent experiment uses the non-homogeneous model (Section 3.3), where per-ray evidence strength decays with distance and incidence angle, to demonstrate that BetP-matched per-observation equivalence holds under spatially varying sensor confidence. The multi-robot simulation and real-data experiments use constant sensor parameters to isolate the accumulation-rule effect from sensor-model heterogeneity. Because the non-homogeneous model attenuates evidence for distant or oblique rays, its average per-ray contribution is weaker than under the constant model, narrowing the Bayesian–DS separation. An ablation that isolates this factor—replacing the non-homogeneous model with the constant model in the single-agent setting, holding the environment, trajectory, and random seeds fixed—increases the boundary-sharpness Bayesian–DS separation by a factor of 1.7 (from 0.0102 to 0.0170). Sensor non-homogeneity thus accounts for part of the smaller single-agent separations, closing roughly an eighth of the single-agent-to-multi-robot boundary-sharpness gap; the remainder reflects environment size, robot count, and alignment regime (full ablation table in Supplementary Material, Section S7.5).

### 4.1. Single-Agent Simulation Setup

The single-agent experiment uses procedurally generated 2D environments of 50 × 50 m at 0.1 m grid resolution (500 × 500 cells). Each environment contains 3 rooms connected by 4 corridors, 5 static obstacles (convex polygons), and 3 dynamic obstacles moving at 0.5 m/s. A single robot follows a deterministic counter-clockwise perimeter patrol trajectory (step size ≈ 0.38 m) with a simulated lidar sensor: 180 rays, 360° field of view (2° angular spacing), 15 m maximum range, Gaussian range noise $\sigma_{r}=0.02$ m.

The sensor model is non-homogeneous (Section 3.3), with base parameters $l_{\mathrm{occ}}=2.0$, $l_{\mathrm{free}}=-0.5$ and decay rates $\lambda_{d}=0.1$, $\lambda_{\alpha }=0.5$. The belief function arm uses pignistic-matched masses derived from these log-odds at each (d,α) pair. The Bayesian arm clamps at $L_{\max}=10$. Each condition is repeated for 15 independent runs with seeds 42–56.

### 4.2. Real Data: Intel Lab and Freiburg 079

#### 4.2.1. Intel Research Lab (Dataset)

The Intel Research Lab dataset [23] contains laser scans collected by a mobile robot in an office environment with furniture, narrow passages, and non-convex obstacles. The sensor is a SICK LMS200 lidar with 180 beams and 180° field of view. We set the effective maximum range to 8.0 m, discarding returns beyond this threshold to match the indoor operating regime.

Scans are split 80%/20% into training and test sets. The training set is used for map construction (both Bayesian and belief function arms process identical scan sequences); the test set generates ground truth occupancy labels for evaluation. This train/test split ensures that ground truth is independent of the mapping data, avoiding circular evaluation.

Scan-split fusion conditions are created by splitting the training scans into R∈{2,4} interleaved subsequences, each assigned to a separate virtual robot. Individual maps are constructed independently and then fused cell-wise, identical to the simulation multi-robot protocol. Real-data scan-split conditions test sensitivity to data partitioning under ideal alignment, not end-to-end multi-robot SLAM performance. This procedure eliminates alignment uncertainty and coverage heterogeneity present in true multi-robot deployments.

Sensor parameters ($l_{\mathrm{occ}}=2.0$, $l_{\mathrm{free}}=-0.5$) are identical to the simulation experiments; belief function masses are derived via the pignistic transform at each observation.

#### 4.2.2. Freiburg Building 079 (Dataset)

The Freiburg 079 dataset provides laser scans from a Pioneer 2 robot equipped with a SICK LMS200 (360 beams, 180° field of view) in a multi-room university building. Processing follows the same protocol as Intel Lab: 0.1 m grid resolution, 8.0 m effective maximum range, 80/20 train/test split, sensor parameters $l_{\mathrm{occ}}=2.0$, $l_{\mathrm{free}}=-0.5$, and scan-split fusion for R∈{2,4}.

Both datasets are standard benchmarks in the occupancy grid mapping literature. Their age (2003–2004) does not affect the validity of the comparison, as both fusion arms process identical sensor data. Ground truth covers approximately 13–14% of cells (approximately 13% for Intel Lab, 14% for Freiburg; occupied/free labels from test scans); frontier and unobserved cells are excluded from evaluation. Generalization to outdoor or 3D lidar datasets is acknowledged as future work.

### 4.3. Metrics

Four complementary metrics are evaluated. For all metrics, the evaluation set $C_{\mathrm{eval}}$ contains cells with at least three test-set observations, ensuring reliable ground truth labeling. Higher values of cell accuracy and boundary sharpness indicate better performance; lower values of Brier score and map entropy indicate better performance.

#### 4.3.1. Cell Accuracy

The fraction of evaluated cells whose thresholded occupancy probability (${\hat{p}}_{c}>0.5$ mapped to occupied) agrees with the binary ground truth label $g_{c}\in \left\{0,1\right\}$. Here, ${\hat{p}}_{c}$ is the Bayesian posterior or BetP(O) for belief functions.

#### 4.3.2. Boundary Sharpness

The mean gradient magnitude at occupied/free transitions, reflecting map utility for path planning (sharper boundaries reduce required safety margins):

(7) $$\mathrm{BdrySharp}=\frac{1}{|C_{\mathrm{bdry}}|}\sum_{c\in C_{\mathrm{bdry}}}∥\nabla {\hat{p}}_{c}∥,$$

where $C_{\mathrm{bdry}}\subset C_{\mathrm{eval}}$ is the set of cells adjacent to a ground truth occupancy transition. Higher sharpness indicates crisper boundary representation.

#### 4.3.3. Brier Score

Mean squared error between predicted probabilities and binary ground truth:


(8) $$\mathrm{Brier}=\frac{1}{|C_{\mathrm{eval}}|}\sum_{c\in C_{\mathrm{eval}}}{\left({\hat{p}}_{c}-g_{c}\right)}^{2}.$$

#### 4.3.4. Map Entropy

Mean per-cell binary entropy, measuring decisiveness:


(9) $$H=-\frac{1}{|C_{\mathrm{eval}}|}\sum_{c\in C_{\mathrm{eval}}}\left[{\hat{p}}_{c}\log_{2}{\hat{p}}_{c}+\left(1-{\hat{p}}_{c}\right)\log_{2}\left(1-{\hat{p}}_{c}\right)\right].$$

A downstream A* path-planning assessment—including a clearance-disagreement check—is reported in the Conclusion (Section 7).

### 4.4. Statistical Analysis Framework

For simulation experiments (15 paired runs), we report mean paired differences with 90% CIs, directional consistency k/N (15/15 has binomial $p=3.1\times 10^{-5}$), and TOST equivalence testing [24,25] with nominal margins δ=0.02 (cell accuracy), 0.03 (boundary sharpness), 0.01 (Brier score), and 0.02 (map entropy). Each margin δ defines the threshold within which absolute differences are considered too small to be of practical concern at the relevant physical scale (e.g., δ=0.02 for cell accuracy corresponds to approximately 2 misclassified cells per 100 evaluated cells at 0.1 m resolution; see Supplementary Material, Section S1.2). These margins are domain-informed rather than calibrated against a specific downstream task; see Section 6.4.5 for further discussion. Holm–Bonferroni correction [26] controls the family-wise error rate. For real-data experiments, spatial block bootstrap (10,000 iterations) provides 95% CIs accounting for spatial autocorrelation. Full statistical details are in Supplementary Material.

The simulation and experimental software used to generate and analyze the reported data was developed with the assistance of an AI coding tool (Claude Code, Anthropic) for drafting and refactoring; all such code was reviewed, tested, and validated by the authors before use. Tool details are given in the Acknowledgments.

## 5. Results

We present results in order of increasing complexity: single-agent simulation, real-data validation, and sensitivity to the matching criterion. Multi-robot simulation results, mechanism tests, and ablation studies are in the Supplementary Material.

### 5.1. Single-Agent Simulation

The single-agent experiment (Section 4.1) compares Bayesian log-odds and DS fusion over 15 independent runs in procedurally generated 50 × 50 m environments with dynamic obstacles.

**Figure 1:**
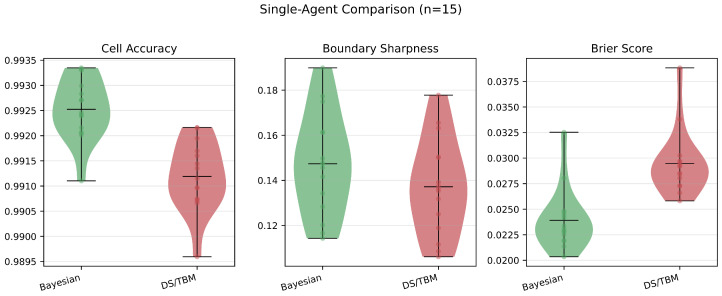
Single-agent comparison: violin plots across 15 runs. Bayesian is directionally favored on all metrics (15/15); absolute differences are 0.001–0.010 on [0, 1] scales.

Bayesian is directionally favored on all three metrics in all 15 runs (15/15, $p=3.1\times 10^{-5}$). Cell accuracy difference is +0.0013 (90% CI [+0.0013,+0.0014]), boundary sharpness +0.0102 ([+0.0096,+0.0108]), and Brier score −0.0056 ([−0.0057,−0.0054]). All three are TOST-equivalent at nominal margins (δ = 0.02, 0.03, 0.01, respectively). The differences are small in absolute terms (<1% on [0,1] scales); see Figure 1.

Despite directional consistency and large Cohen’s *d* values (d=4.6–14.9), the absolute differences are below all TOST equivalence margins. The large effect sizes reflect very low within-method variance ($s_{d}=0.0001$–0.014) inherent to controlled computational experiments, not substantively different map quality (Supplementary Material, Section S1.3).

### 5.2. Multi-Robot Results

Multi-robot simulation (3 robots, PGO alignment, 2 conditions) shows the same directional pattern with larger separations on boundary sharpness and map entropy (Table 1). These separations are not TOST-equivalent at nominal margins. Full multi-robot results, a mechanism test across R=1–5 robots, and ablation studies are in Supplementary Material. The $L_{\max}$ ablation (Section S7.1) shows that the directional finding persists even without clamping ($L_{\max}=\infty$, 15/15 consistency). Replacing Dempster’s rule with Yager’s rule (Section S7.2) worsens boundary sharpness by 21–25% because conflict-to-ignorance transfer preserves excessive boundary uncertainty. A DS regularization floor $m_{OF,\min}\in \left\{0.001,0.01,0.05\right\}$ (Section S7.3) does not close the gap. Sensor parameter sensitivity (Section S7.4) is discussed in Section 3.3. The multi-robot simulation deliberately uses ground-truth pose graph optimization (PGO) alignment as an experimental control: by eliminating pose alignment error, the design isolates the fusion-rule effect from alignment noise, providing an estimate of the separation attributable to the accumulation rule alone under conditions where pose error is removed as a confound. Full details of the multi-robot protocol are in Supplementary Material, Section S5.

### 5.3. Sensitivity to Matching Criterion

When per-observation equivalence uses normalized plausibility $P_{\mathrm{Pl}}$ instead of BetP (single-agent only, 15 seeds), the direction reverses: DS achieves better boundary sharpness (Δ=−0.002, 15/15) and Brier score (Δ=+0.008, 15/15), while cell accuracy remains tied (|Δ|<0.0001). The reversal is mechanistically expected: $P_{\mathrm{Pl}}$ matching allocates 44% less ignorance mass per observation than BetP matching (Section 3.2), producing sharper DS updates that converge faster at boundary cells.

Under the tested conditions, the ranking of fusion rules depends on the probability transform chosen for sensor model matching, not solely on the accumulation mechanism. The present study adopts BetP as the matching criterion because it is the standard decision-theoretic choice within the TBM framework [9] and the most widely used transform in the evidential occupancy-grid literature. However, the reversal observed under $P_{\mathrm{Pl}}$ matching demonstrates that the transform choice is not neutral: conclusions drawn under BetP do not automatically transfer to alternative transforms. Extending the $P_{\mathrm{Pl}}$ check to the multi-robot experiment (both noise conditions, 15 seeds), the BetP boundary-sharpness advantage for Bayesian (Δ≈+0.06 to +0.07) collapses to within the equivalence margin under $P_{\mathrm{Pl}}$: a near-tie under the dynamic-baseline condition (Δ=+0.006) and a slight DS advantage under elevated noise (Δ=−0.007). The boundary-sharpness ranking is thus transform-determined in the multi-robot setting as well as the single-agent setting, and the multi-robot separations reported under BetP do not persist under $P_{\mathrm{Pl}}$. Extension of the $P_{\mathrm{Pl}}$ analysis to the real-data experiments remains future work.

### 5.4. Real-Data Validation

#### 5.4.1. Intel Research Lab

On Intel Lab [23], the Bayesian arm is favored on cell accuracy (+0.018 to +0.022 across scan-split configurations; 95% spatial block bootstrap CIs exclude zero) and Brier score (−0.013 to −0.017; CIs exclude zero). Boundary sharpness differences are not statistically significant (all CIs include zero), consistent with the $L_{\max}$ ablation (Supplementary Material) attributing boundary differences to clamping artifacts.

#### 5.4.2. Freiburg Building 079

On Freiburg 079 [27], Bayesian is favored on all three metrics: cell accuracy +0.007, boundary sharpness +0.010 to +0.011, Brier score −0.007 (all CIs exclude zero across all scan-split configurations).

##### Real-Data Summary

**Figure 2:**
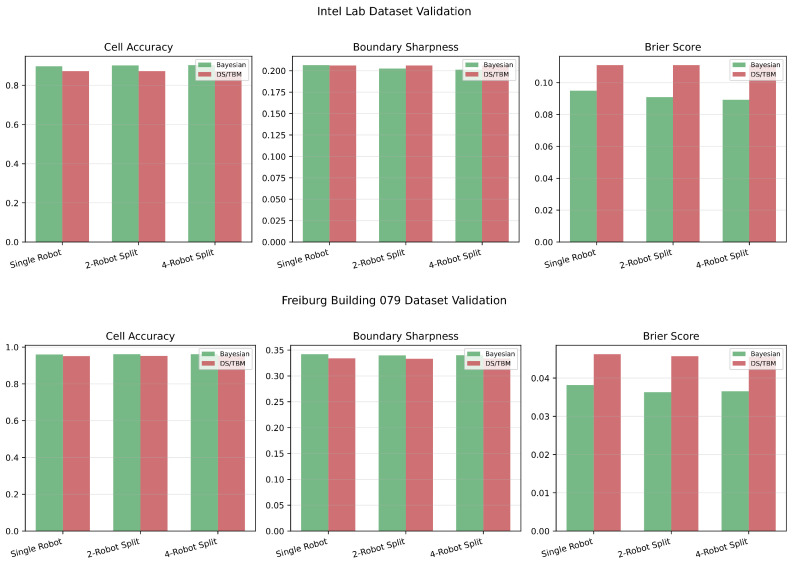
Real-data validation. *Top:* Intel Lab—cell accuracy and Brier score favor Bayesian; boundary sharpness is not statistically significant. *Bottom:* Freiburg 079—all three metrics favor Bayesian. Both datasets use BetP-matched point-probability evaluation across scan-split configurations.

On both datasets (Figure 2), the BetP-matched simulation direction is preserved for cell accuracy and Brier score. Boundary sharpness favors Bayesian on Freiburg 079 but is non-significant on Intel Lab. These are BetP-specific point-probability evaluations with uncalibrated sensor parameters; they confirm the directional finding under the same matching protocol, not independent generalization.

## 6. Discussion

### 6.1. The Sensor Model Confound

The confound demonstration (Section 3.1) shows that independent sensor parameterization—representative of community defaults—can reverse the apparent direction of a cross-framework comparison. This finding does not imply errors in prior work; the confound was not previously recognized. It does suggest that previously reported DS advantages [10,11] may be partly attributable to unmatched parameterization. We restrict this observation to our own experiment; we did not reproduce prior parameterizations. The pignistic matching methodology provides a systematic solution for future comparisons.

### 6.2. Transform Dependence

The sensitivity analysis in Section 5.3 demonstrates that the ranking of fusion rules is not invariant to the probability transform used for sensor model matching: the direction reverses on boundary sharpness and Brier score when $P_{\mathrm{Pl}}$ replaces BetP. The observed differences therefore cannot be attributed to the accumulation rule alone. The scope limitations arising from this transform dependence are discussed in Section 6.4.

### 6.3. Conflict Level and Metric Divergence

The accumulation divergence described in Section 3.4 predicts that metric differences concentrate at boundary cells, where conflicting observations drive Dempster’s 1/(1−K) normalization to converge more slowly than additive log-odds. Consistent with this mechanism—though also with the larger dynamic range of boundary-focused metrics—the largest Bayesian–DS separations appear on boundary sharpness in simulation experiments, while cell accuracy, which averages over all evaluated cells, shows the smallest separations there (Table 1; Figure 1). In the multi-robot simulation, conflict is concentrated at a small minority of boundary cells (4.7% under the dynamic-baseline condition and 7.6% under elevated sensor noise); among those, the median Dempster conflict is K≈0.07–0.14 (per-condition statistics in Supplementary Material, Section S5). The conflict-normalization effect is most cleanly isolated in simulation, where ground-truth alignment removes pose error; on the real datasets the two frameworks are practically equivalent on boundary sharpness (Table 1), consistent with this being one of several small effects rather than a dominant factor in real deployments.

### 6.4. Limitations

#### 6.4.1. Point-Probability Evaluation Only

All metrics operate on point probabilities (BetP or σ(L)). The belief function interval [Bel(A),Pl(A)]—a distinctive feature of the framework—is not evaluated. The interval width $\mathrm{Pl}\left(O\right)-\mathrm{Bel}\left(O\right)=m_{OF}$ carries information absent from the Bayesian representation; our point-probability evaluation does not access this information, and the conclusions of this paper apply only to the point-probability projection. The downstream consequences of this evaluation choice are discussed in Section 6.4.4.

#### 6.4.2. BetP-Conditional Comparison

The matching equates pignistic probabilities, not the belief function representations themselves. This restricts DS to operating in a regime where it is most similar to a probability model. A comparison that preserves the full mass-function representation would require fundamentally different evaluation metrics.

As shown by the $P_{\mathrm{Pl}}$ sensitivity analysis (Section 5.3), the choice of probability transform is itself a scope limitation: the BetP-based ranking does not automatically extend to other transforms. The $P_{\mathrm{Pl}}$ check has been run in both the single-agent and multi-robot settings (Section 5.3); alternative transforms (e.g., maximum entropy) and extension to the real-data experiments remain future work.

#### 6.4.3. Real-Data Scope

Both datasets use early-2000s indoor SICK LMS200 lidar. Validation on modern multi-beam sensors (e.g., Newer College Dataset with Ouster OS1-64, or KITTI-360 indoor sequences) would test generalization to higher-density point clouds. Scan-split conditions use ideal alignment, not true multi-robot deployment. Extending the multi-robot evaluation to noisy PGO alignment from a realistic SLAM back-end would quantify how localization uncertainty interacts with the fusion-rule differences observed under ideal conditions. Sensor parameters ($l_{\mathrm{occ}}=2.0$, $l_{\mathrm{free}}=-0.5$) are inherited from the simulation experiments rather than fitted to the SICK LMS200 beam characteristics. Because the DS masses are derived via the pignistic transform at each observation, per-observation BetP equivalence holds regardless of the absolute parameter values, so the matching protocol remains valid. However, calibrated parameters could shift the absolute metric values and the magnitude of Bayesian–DS separation; a future sensor-specific calibration step using real-data log-likelihood optimization would quantify this sensitivity.

#### 6.4.4. Downstream Evaluation Limitations

Practical significance is assessed via A* path planning only—a deterministic shortest-path task for which Bayesian point estimates suffice. The interval-valued features that belief functions provide—notably the [Bel(A),Pl(A)] uncertainty interval and the inter-source conflict measure *K*—are not exercised by A*, which consumes only a single occupancy threshold. This biases the downstream evaluation toward scenarios in which the two frameworks are expected to perform equivalently. Downstream tasks that could expose differential performance include risk-aware path planning (exploiting Bel/Pl intervals for conservative traversability), frontier-based exploration (using m(Θ) as an uncertainty-driven exploration target), and dynamic-obstacle detection (using conflict *K* as an anomaly indicator). These directions remain untested and represent the principal limitation of the present evaluation.

#### 6.4.5. Statistical Design

TOST margins are not grounded in formal downstream task performance thresholds. Real-data tests are not pre-registered; they confirm the simulation direction under the same evaluation protocol, not independent generalization.

## 7. Conclusions

Cross-framework comparison of Bayesian and belief function occupancy grid fusion is sensitive to sensor model parameterization: without per-observation matching, independent parameterization from community defaults reversed boundary sharpness by 23–36 percentage points across two noise conditions in our experiments (Section 3.1). We develop a pignistic-transform-based matching methodology that controls for the sensor-model confound by equating per-observation decision probabilities.

Under the tested BetP-matched point-probability evaluation, the two frameworks produce practically equivalent maps, with a small directional advantage for Bayesian log-odds in single-agent simulation and on both real datasets (absolute differences 0.001–0.022 on [0,1] scales). Cohen’s *d* values, while large, reflect very low within-method variance rather than substantively different absolute performance (see Supplementary Material, Section S1.3). A downstream A* path-planning assessment (500 start–goal pairs; protocol in Supplementary Material, Section S9) shows 100% shared reachability and 77% path equivalence between the two arms, with a mean path-length difference of 0.08 cells, consistent with practical equivalence for shortest-path navigation. A residual 7% of pairs show a safety-critical clearance disagreement, indicating that this equivalence is specific to shortest-path length and does not automatically extend to clearance-sensitive tasks. Under $P_{\mathrm{Pl}}$ matching, the direction reverses for boundary sharpness and Brier score: the observed ranking depends on the matching transform, not solely on the accumulation mechanism.

These findings apply to point-probability metrics on 2D binary grids under Dempster’s and Yager’s combination rules; the interval-valued belief-plausibility representation remains unevaluated. Future work should extend to modern lidar datasets, calibrated real-sensor models, alternative combination rules, and interval-valued evaluation. Extension to 3D voxel grids is feasible for binary occupancy [28] but multi-class semantic grids pose a combinatorial challenge: the DS frame of discernment grows to $2^{K}$ focal elements for *K* classes, making naive fusion intractable. Current approaches restrict belief assignments to singleton hypotheses only [29,30], reducing storage to *K* masses per cell at the cost of discarding partial-set uncertainty—a key representational advantage that belief functions offer over Bayesian models. Whether the matching methodology developed here transfers to these settings remains an open question.

## Figures and Tables

**Table 1:** Cross-environment summary: mean paired difference (Bayesian − DS). Simulation reports paired Δ over 15 runs with directional consistency *k*/*N*; real data reports ranges across scan-split configurations with 95% spatial block bootstrap CIs.

Environment	Metric	Δ	Evidence
Single-agent (15 runs)	Cell acc.	+0.001	15/15
	Bdry sharp.	+0.010	15/15
	Brier score	−0.006	15/15
Multi-robot, dynamic	Cell acc.	+0.011	15/15
	Bdry sharp.	+0.070	15/15
	Map entropy	−0.026	15/15
Multi-robot, noisy	Cell acc.	+0.009	15/15
	Bdry sharp.	+0.063	15/15
	Map entropy	−0.026	15/15
Intel Lab (*R* = 2, 4)	Cell acc.	+0.018 to +0.022	CI excl. 0
	Bdry sharp.	—	n.s.
	Brier score	−0.013 to −0.017	CI excl. 0
Freiburg 079 (*R* = 2, 4)	Cell acc.	+0.007	CI excl. 0
	Bdry sharp.	+0.010 to +0.011	CI excl. 0
	Brier score	−0.007	CI excl. 0

## Data Availability

All experiment code, sensor model implementations, and configuration files for reproducing the reported experiments are openly available in a Zenodo reproducibility package (DOI: https://doi.org/10.5281/zenodo.20476371) under the MIT license. The Intel Research Lab dataset is publicly available [23]. The Freiburg Building 079 dataset is publicly available [27]. All simulation experiments use fixed random seeds (42–56) and produce deterministic results.

## Supplementary Materials

The following supporting information can be downloaded at: https://www.mdpi.com/article/10.3390/s26134266/s1, Supplementary material contains: proofs (single-observation equivalence, $m_{OF}$ monotonic decay, closed-form accumulation), multi-robot simulation results, mechanism test (R=1–5), $L_{\max}$, sensor-parameter, and sensor non-homogeneity ablations, Yager’s rule and DS regularization controls, statistical methodology details (TOST, effect sizes, multiplicity correction, spatial block bootstrap, CI verification) [31,32,33], convergence analysis, additional theoretical results (bounded-associative-additive trilemma, sufficient-statistic characterization), and a downstream path-planning evaluation protocol.
