# Supplementary material for: Sensor-Model Matching for Controlled Comparison of Bayesian and Belief-Function Occupancy Grid Fusion
Source: Sensors (Basel). 2026 Jul 4;26(13):4266. doi: 10.3390/s26134266 (PMC13363857; doi:10.3390/s26134266)
Supplement: Supplementary file 1 [file sensors-26-04266-s001.zip › sensors-4295652-supplementary.pdf]

Supplementary Material: Sensor-Model Matching for Controlled Comparison of Bayesian and Belief-Function Occupancy Grid Fusion

Tatiana Berlenko, Kirill Krinkin

This supplement accompanies the main paper and is organized as follows: Section S1 presents statistical methodology details. Section S2 contains supporting proofs. Section S3 provides convergence analysis. Section S4 presents prior-work detailed comparison. Section S5 reports multi-robot simulation results. Section S6 reports the mechanism test. Section S7 reports ablation studies. Section S8 presents additional theoretical results. Section S9 documents the downstream path-planning evaluation protocol.

S1 Statistical Methodology

This section presents the complete statistical analysis framework summarized in the main text.

S1.1 Directional Analysis

As a distribution-free primary measure of effect reliability, we report k/N: the number of runs in which the Bayesian arm achieves a higher (or lower, for Brier score and entropy) metric value than the belief function arm. This sign-test-like statistic requires no assumptions about effect size distributions and is robust to outliers.

S1.2 Equivalence Testing (TOST)

We apply the Two One-Sided Tests (TOST) procedure Schuirmann [1987], Lakens [2017] to assess whether performance differences fall within practically negligible bounds. TOST rejects the null hypothesis of non-equivalence when the $(1-2\alpha)$ confidence interval for the mean paired difference $\bar{d} = \overline{x-y}$ falls entirely within $(-\delta, +\delta)$, where δ is the equivalence margin.

Rather than reporting equivalence at a single pre-specified margin, we present a **sensitivity analysis** as the primary TOST result: for each metric–condition pair, we sweep the margin from $0.5\times$ to $2\times$ the nominal value and report the smallest margin at which equivalence holds and the breakpoint at which it fails.

Nominal margins: $\delta = 0.02$ (cell accuracy), 0.03 (boundary sharpness), 0.01 (Brier score), 0.02 (map entropy). At 0.1 m grid resolution, a cell accuracy margin of $\delta = 0.02$ corresponds to approximately 2 misclassified cells per 100 observed cells.

S1.3 Effect Sizes

Paired Cohen's d is computed as $d = \bar{d}/s_d$, where s_d is the standard deviation of the paired differences. Confidence intervals use the Hedges–Olkin Hedges and Olkin [1985] variance approximation:

$$\widehat{\mathrm{Var}}(d) = \frac{1}{n} + \frac{d^2}{2(n-1)}. \tag{S1}$$

Interpreting large d values In computational experiments with controlled environments and fixed random seeds, within-run variance approaches zero, inflating d without limit. The effect sizes reported ($d = 4.6$–14.9) reflect very low within-method variance ($s_d = 0.0001$–0.014), not large absolute differences. Raw mean differences are 0.001–0.070 on $[0, 1]$ scales.

S1.4 Multiplicity Correction

The nine simulation TOST comparisons (3 metrics × 3 conditions: single-agent, dynamic baseline, noisy sensor) are corrected as a single family using the Holm–Bonferroni step-down procedure Holm [1979].

S1.5 Real-Data Statistical Analysis

The real-data experiments produce a single map per condition. We use a *spatial block bootstrap* ($B = 10$ cells $= 1.0$ m, 10,000 iterations) to construct 95% CIs accounting for spatial autocorrelation. A sensitivity analysis over $B \in \{5, 10, 20\}$ confirms stable verdicts (section S1.8).

S1.6 Bayes Factors

As a complementary measure, we compute BF_{01} using a conjugate normal model with scale $= \delta$. All five TOST-equivalent comparisons yield $\mathrm{BF}_{01} > 10^6$; all four non-equivalent yield $\mathrm{BF}_{01} \approx 0$. Results are fully consistent with frequentist TOST verdicts.

S1.7 CI Verification

Table S1 compares Hedges–Olkin CIs against exact non-central t CIs Steiger [2004] for all nine simulation comparisons. Maximum absolute discrepancy is 0.59 (< 4% of point estimate). All qualitative conclusions are identical.

Table S1: Cohen's d CI verification: Hedges–Olkin (1985) approximation vs. exact non-central t inversion (Steiger, 2004). Maximum discrepancy is 0.59, confirming qualitative conclusions are unaffected.

Experiment	Metric	d	HO 95% CI	NCT 95% CI	Disc.
Single-agent	Cell Accuracy	+13.51	[+8.01, +19.02]	[+8.54, +18.48]	0.54
	Boundary Sharpness	+7.78	[+4.58, +10.98]	[+4.88, +10.67]	0.31
	Brier Score	−14.93	[−21.00, −8.85]	[−20.41, −9.44]	0.59
Multi-robot (dyn.)	Cell Accuracy	+6.14	[+3.59, +8.69]	[+3.83, +8.44]	0.25
	Boundary Sharpness	+5.96	[+3.48, +8.44]	[+3.71, +8.19]	0.24
	Map Entropy	−7.11	[−10.04, −4.17]	[−9.75, −4.45]	0.29
Multi-robot (noisy)	Cell Accuracy	+6.03	[+3.52, +8.53]	[+3.76, +8.29]	0.25
	Boundary Sharpness	+4.62	[+2.67, +6.57]	[+2.85, +6.38]	0.19
	Map Entropy	−6.32	[−8.94, −3.70]	[−8.69, −3.95]	0.26

S1.8 Block Size Sensitivity

Table S2 reports CIs at $B \in \{5, 10, 20\}$ for both real datasets. All significance verdicts are stable.

Table S2: Block size sensitivity for spatial block bootstrap CIs. Each cell shows the 95% CI for the metric delta (Bayesian − Dempster). All significance verdicts are stable across block sizes.

Dataset	Setup	Metric	B=5 (0.5 m)	B=10 (1.0 m)	B=20 (2.0 m)
Intel Lab	1-source	Cell Accuracy	[+0.014, +0.021]	[+0.014, +0.022]	[+0.013, +0.023]
		Boundary Sharpness	[−0.006, +0.007]	[−0.007, +0.007]	[−0.007, +0.007]
		Brier Score	[−0.016, −0.010]	[−0.016, −0.010]	[−0.016, −0.009]
	2-way split	Cell Accuracy	[+0.018, +0.024]	[+0.017, +0.025]	[+0.017, +0.026]
		Boundary Sharpness	[−0.010, +0.003]	[−0.010, +0.003]	[−0.011, +0.004]
		Brier Score	[−0.018, −0.014]	[−0.019, −0.013]	[−0.020, −0.013]
	4-way split	Cell Accuracy	[+0.020, +0.025]	[+0.019, +0.026]	[+0.018, +0.027]
		Boundary Sharpness	[−0.012, +0.001]	[−0.013, +0.002]	[−0.013, +0.003]
		Brier Score	[−0.019, −0.015]	[−0.020, −0.014]	[−0.021, −0.014]
Freiburg 079	1-source	Cell Accuracy	[+0.005, +0.009]	[+0.005, +0.009]	[+0.005, +0.009]
		Boundary Sharpness	[+0.005, +0.018]	[+0.005, +0.018]	[+0.005, +0.018]
		Brier Score	[−0.009, −0.006]	[−0.009, −0.005]	[−0.009, −0.005]
	2-way split	Cell Accuracy	[+0.005, +0.009]	[+0.005, +0.009]	[+0.005, +0.010]
		Boundary Sharpness	[+0.005, +0.016]	[+0.005, +0.016]	[+0.005, +0.016]
		Brier Score	[−0.009, −0.006]	[−0.009, −0.006]	[−0.010, −0.005]
	4-way split	Cell Accuracy	[+0.005, +0.009]	[+0.005, +0.009]	[+0.005, +0.010]
		Boundary Sharpness	[+0.004, +0.016]	[+0.004, +0.016]	[+0.004, +0.016]
		Brier Score	[−0.009, −0.006]	[−0.009, −0.005]	[−0.009, −0.005]

S2 Supporting Proofs

S2.1 Single-Observation Pignistic Equivalence

The proof of single-observation pignistic equivalence is given in the main text (Theorem 1, Section 3.2).

S2.2 Proof of Closed-Form Accumulation

Under vacuous prior and N identical consonant occupied observations with $m_{\text{obs}} = (a, 0, 1-a)$:

Proof. By induction on N. Base $N = 1$: vacuous identity gives the observation mass. Step: assume $m_O^{(N)} = 1 - (1-a)^N$, $m_{OF}^{(N)} = (1-a)^N$. Since $m_F^{(N)} = m_{F,\text{obs}} = 0$, conflict $K = 0$. Dempster combination: $m_O^{(N+1)} = m_O^{(N)} + a(1-a)^N = 1-(1-a)^{N+1}$. $m_{OF}^{(N+1)} = (1-a)^N(1-a) = (1-a)^{N+1}$. □

S2.3 Proof of m_{OF} Monotonic Decay

Theorem S2.1 (m_{OF} monotonic decay)**.** *Let m^{pr} have $m_{OF}^{\text{pr}} > 0$ and let m^{obs} be non-degenerate ($0 < m_{OF}^{\text{obs}} < 1$). Then $m_{OF}^{\text{post}} < m_{OF}^{\text{pr}}$.*

Proof. The posterior ignorance mass is $m_{OF}^{\text{post}} = m_{OF}^{\text{pr}} \cdot m_{OF}^{\text{obs}}/(1-K)$. It suffices to show $m_{OF}^{\text{obs}} < 1 - K$. Expanding:

$$1 - K - m_{OF}^{\text{obs}} = m_O^{\text{obs}}(m_O^{\text{pr}} + m_{OF}^{\text{pr}}) + m_F^{\text{obs}}(m_F^{\text{pr}} + m_{OF}^{\text{pr}}).$$

Since the observation is non-degenerate, at least one of $m_O^{\text{obs}}, m_F^{\text{obs}} > 0$. Since $m_{OF}^{\text{pr}} > 0$, both coefficients are positive. Hence $1 - K - m_{OF}^{\text{obs}} > 0$. □

S3 Convergence Analysis

Table S3 compares the two accumulation trajectories for N identical occupied observations.

Table S3: Convergence comparison ($l_{\text{occ}} = 2.0$, $L_{\max} = 10$, $a = 0.7616$). At $N = 1$ the methods agree by construction. Bayesian clamp saturates at $N = 5$; DS overtakes at $N \approx 7$.

N	L	$p(O)$	m_O	m_{OF}	BetP(O)	BetP $- p$
1	2.0	0.8808	0.7616	0.2384	0.8808	+0.0000
2	4.0	0.9820	0.9432	0.0568	0.9716	−0.0104
5	10.0	0.999955	0.9992	0.0008	0.9996	−0.0003
10	10.0	0.999955	≈ 1	$< 10^{-6}$	≈ 1	+0.00005

S4 Prior-Work Detailed Comparison

In the credibilist grid literature Moras et al. [2011b,a], log-odds parameters and belief function masses are set independently. In vinySLAM Huletski et al. [2017], the TBM cell model and scan matching cost function were changed simultaneously without ablation. In Nuss et al. Nuss et al. [2018], the RFS-based grid and the Bayesian baseline use different observation models. The common pattern is the absence of a per-observation matching criterion.

S5 Multi-Robot Simulation Results

Protocol. Each run places 3 robots in a 20×20 m environment (0.1 m resolution, single room, 5 static obstacles). The robots start at perimeter fractions $\{0.0, 0.02, 0.97\}$ and follow fixed patrol trajectories of 200 steps each, subject to per-step translational drift $\sigma = 0.02$ m and angular drift $\sigma = 0.005$ rad. The simulated LiDAR has an 8 m maximum range, 90 rays, and range noise $\sigma = 0.03$ m. Two conditions are run, 15 runs each (seeds 42–56): *dynamic baseline* (3 moving obstacles at 0.3 m/s) and *noisy sensor* (no dynamic obstacles; false-positive rate 0.05, false-negative rate 0.02). Each robot maintains its own occupancy grid; the grids are registered by ground-truth pose-graph-optimization (PGO) alignment (rendezvous distance 2.5 m) and fused cell-wise. Ground-truth alignment is a deliberate experimental control that removes pose-estimation error, so the comparison isolates the accumulation-rule effect. The DS/TBM arm uses pignistic-matched masses derived from the Bayesian log-odds ($l_{\text{occ}} = 2.0$, $l_{\text{free}} = -0.5$), so per-observation BetP equivalence holds.

Bayesian is directionally favored on all metrics in both conditions (15/15). Cell accuracy differences are TOST-equivalent at $\delta = 0.02$; boundary sharpness and map entropy are not equivalent at nominal margins (see Table 1 in the main paper for summary).

Full per-condition details: dynamic baseline cell accuracy $\Delta = +0.011$, $d = +6.14$; noisy sensor cell accuracy $\Delta = +0.009$, $d = +6.03$. Boundary sharpness $\Delta = +0.063$–$+0.070$, not equivalent. Map entropy $\Delta = -0.026$, not equivalent.

Sensor-model matching and the boundary-sharpness reversal. The boundary-sharpness reversal that motivates per-observation matching (Section 3.1 of the main paper) is summarized per condition in table S4. Under community-default DS masses (0.75/0.10/0.15), belief-function fusion holds a boundary-sharpness advantage over the Bayesian arm (+14% under the dynamic-baseline condition, +6% under elevated sensor noise); under pignistic-matched masses the same comparison reverses to a deficit (−22% and −17%, respectively). Within each condition the Bayesian arm is identical across the two parameterizations—only the DS masses change—so the sign flip is a clean paired effect of the sensor model, not of the fusion rule.

Boundary-cell conflict. table S5 reports the Dempster conflict mass at boundary cells for the matched runs, underlying the conflict-normalization discussion in the main paper (Section 6.3).

Table S4: Boundary-sharpness reversal under community-default versus pignistic-matched DS masses (3 robots, 15 runs each, seeds 42–56). The relative column is (DS − Bayesian)/Bayesian, the belief-function (DS) arm relative to the Bayesian arm: it is positive exactly when the DS column exceeds the Bayesian column (a belief-function advantage) and negative otherwise (a deficit). The Bayesian baseline is identical between the two parameterizations within each condition, isolating the sensor-model effect. The matched absolute differences (Bayesian − DS = +0.070 for dynamic, +0.063 for noisy) appear in Table 1 of the main paper; see also Section 3.1.

Condition	DS masses	Bayesian	DS	Relative
Dynamic baseline	Default	0.3135	0.3560	+14%
Dynamic baseline	Matched	0.3135	0.2435	−22%
Elevated noise	Default	0.3724	0.3953	+6%
Elevated noise	Matched	0.3724	0.3096	−17%

Table S5: Boundary-cell Dempster conflict in the multi-robot simulation (matched DS arm, 15 runs each, seeds 42–56). $K = m_1(O)\,m_2(F) + m_1(F)\,m_2(O)$ is the unclamped Dempster conflict mass evaluated at static-wall boundary cells (ground truth ≥ 0.5). The *conflicting* column is the fraction of boundary cells with $K > 0.01$; *median* K is taken over those conflicting cells. Each quantity is reduced as the median over the converged tail (steps ≥ 150 of 200) and then across the 15 seeds.

Condition	Conflicting boundary cells	Median K (conflicting)
Dynamic baseline	4.7%	0.074
Elevated noise	7.6%	0.141

Conflict is concentrated at a small minority of boundary cells, and among those cells the median conflict is moderate.

S6 Mechanism Test

To test whether the directional finding persists or reverses as the number of fusion events increases, we run the single-agent experiment with $R \in \{1, 2, 3, 5\}$ robots sharing 500 total trajectory steps. The boundary sharpness advantage is small and stable across robot count, ranging from +0.013 ($R = 1$) to +0.016 ($R = 2$), with no significant trend (linear regression $p = 0.61$). No crossover is observed at any robot count.

S7 Ablation Studies

S7.1 $L_{\max}$ Ablation

The directional finding persists at $L_{\max} = \infty$ with 15/15 consistency.

S7.2 Yager's Rule

Yager's rule Yager [1987] performs worse than Dempster on boundary sharpness (−21% to −25%) because conflict-to-ignorance transfer preserves excessive boundary uncertainty.

S7.3 DS Regularization

A minimum ignorance floor $m_{OF,\min} \in \{0.001, 0.01, 0.05\}$ does not close the gap; on boundary sharpness it widens the disadvantage.

Table S6: $L_{\max}$ ablation: Bayesian minus Dempster delta (15 runs).

Metric	$L_{\max} = 5$	10	20	∞	k/N
Cell accuracy	+0.0013	+0.0013	+0.0013	+0.0013	15/15
Boundary sharpness	+0.007	+0.010	+0.010	+0.010	15/15
Brier score	−0.006	−0.006	−0.006	−0.006	15/15

Table S7: Sensor non-homogeneity ablation: paired Bayesian-minus-DS separation magnitude $|\bar{d}|$ (15 runs, seeds 42–56) under the non-homogeneous (NH) and constant (C) sensor models. The constant model is the multi-robot sensor model. Boundary sharpness is the metric on which the multi-robot experiment reported the larger separations.

Metric	NH $|\bar{d}|$	C $|\bar{d}|$	C / NH
Boundary sharpness	0.0102	0.0170	1.7×
Cell accuracy (ceiling)	0.0013	0.0048	—
Brier score (floor)	0.0056	0.0039	0.7×

S7.4 Sensor Parameter Sensitivity

The directional finding is consistent across four parameterizations (weak, default, strong, symmetric). The advantage is smallest for the symmetric sensor ($|l_{\text{occ}}| = |l_{\text{free}}|$).

S7.5 Sensor Non-Homogeneity Ablation (R2.6)

The single-agent and multi-robot experiments use different sensor models: the single-agent setting applies a non-homogeneous inverse sensor model (per-ray log-odds attenuated by distance and incidence angle, $\lambda_d = 0.1$, $\lambda_\alpha = 0.5$), whereas the multi-robot and real-data settings use a constant model ($l_{\text{occ}} = 2.0$, $l_{\text{free}} = -0.5$, no decay). This raises the question of whether the sensor-model difference, rather than the experimental setting, accounts for the larger Bayesian–DS separations observed in the multi-robot experiment.

Design. We isolate the sensor model as the *sole* varied factor in the single-agent setting. Two levels are compared: (NH) the non-homogeneous model, which produces the paper's official single-agent results; and (C) the constant model, identical to the multi-robot/real-data sensor model. The constant model is the ($d = 0, \alpha = 0$) special case of the same pignistic mass construction, so per-observation BetP equivalence holds at both levels. Environment (50×50 m, 3 rooms, 4 corridors, 5 static and 3 dynamic objects), trajectory (perimeter patrol, 500 steps), LiDAR configuration, fusion arms, and the 15 random seeds (42–56) are held fixed. The committed NH results reproduce bit-for-bit under the current code (seeds 42–44, maximum absolute difference 0.000), confirming no code drift confounds the comparison.

Separation metric. For each map-quality metric we compute the paired Bayesian-minus-DS mean signed difference over the 15 runs and its absolute magnitude $|\bar{d}|$, then compare the two sensor-model levels. Table S7 reports the result.

Result. On boundary sharpness—the metric on which the multi-robot experiment reported the larger separations—replacing the non-homogeneous model with the constant (multi-robot) model increases the single-agent Bayesian–DS separation by a factor of 1.7 ($0.0102 \to 0.0170$). Sensor non-homogeneity is therefore a genuine driver of the smaller single-agent separations. Its contribution is, however, partial: the increment accounts for only $(0.0170 - 0.0102)/(0.070 - 0.0102) \approx 1/8$ of the single-agent to multi-robot boundary-sharpness gap (the multi-robot

boundary-sharpness delta is ≈ 0.070, section S5). The remaining $\approx 7/8$ reflects the other uncontrolled differences between the two settings: environment size, robot count, and the PGO alignment regime.

Scope and honesty constraints. The causal claim is scoped to boundary sharpness and to the *raw separation magnitude*. It does not extend to all metrics. The Brier-score separation moves in the opposite direction (C is 0.7× NH): the constant model delivers stronger average per-ray evidence, which nearly halves the absolute Brier score and hence *improves* calibration for both arms, shrinking the absolute gap—a floor effect, not a reversal of the directional finding. Cell accuracy is at ceiling (≈ 0.993) in both settings and is uninformative here. Finally, the claim is about raw magnitude, not standardized effect size: paired Cohen's d is roughly flat across the two levels (boundary sharpness 7.78 vs. 7.04), because the within-run standard deviation scales with the mean. We therefore report "separation magnitude", not "stronger statistical effect".

S8 Additional Theoretical Results

This section contains theoretical results developed during the research but not required for the main empirical contribution.

S8.1 Bounded-Associative-Additive Trilemma

Theorem S8.1. *For any $C > 0$, there is no function $F\colon [-C, C]^2 \to [-C, C]$ satisfying simultaneously: (B) $F(a, b) \in [-C, C]$ for all a, b; (A) $F(F(a, b), c) = F(a, F(b, c))$ for all a, b, c; (P) $F(a, b) = a + b$ whenever $|a + b| \leq C$.*

Proof. For each $N \geq 2$, set $l = C/N$ and define $\varphi(0) = 0$, $\varphi(n + 1) = F(\varphi(n), l)$. By (P), $\varphi(n) = nl$ for $n \leq N$, so $\varphi(N) = C$. By induction using (A), $F(\varphi(m), \varphi(n)) = \varphi(m + n)$ for $m, n \geq 0$.

Let $v = \varphi(N+1) = F(C, l)$. From the cancellation identity $F(\varphi(m), -l) = \varphi(m-1)$ (proved via (A) and (P)): $F(v, -l) = C$. If $|v - l| \leq C$, then (P) gives $v = C + l > C$, contradicting (B). So $v < -C + l$.

Since $v \in [-C, -C + C/N)$, the recovery phase gives $\varphi(2N) = v + (N - 1)l \in [-C/N, 0)$. By the homomorphism: $F(C, C) = \varphi(2N) \in [-C/N, 0)$ for every $N \geq 2$. As $N \to \infty$, $F(C, C) \geq 0$ and $F(C, C) < 0$ simultaneously. Contradiction. □

This trilemma characterizes three design strategies: clamped Bayesian sacrifices (A), Dempster's rule (operating in a higher-dimensional mass simplex) forgoes (P), and unclamped Bayesian sacrifices (B).

S8.2 Minimal Sufficiency of (L, n)

Under a Binary Symmetric Channel model with known error rate ε, the pair (L, n) (log-odds and observation count) is a minimal sufficient statistic for the true cell state. This follows from Fisher–Neyman factorization Casella and Berger [2002]: the likelihood ratio depends on the data only through $k = n/2 + L/(2l)$. This is a standard exponential-family result; its relevance is that the DS/TBM representation (m_O, m_F, m_{OF}) cannot recover n after moderate accumulation (the sensitivity $(dm_{OF}/dn)^2$ decays exponentially).

S9 Downstream Path-Planning Evaluation

This section documents the protocol behind the downstream A* path-planning sanity check reported in the Conclusion, which probes whether the small map-level differences between the

Bayesian and belief-function (Dempster's rule) arms propagate into the navigation task that occupancy grids are built to serve. The evaluation is fully deterministic and reproducible; the implementation is `src/experiments/downstream_eval/`.

S9.1 Map Source and Obstacle Classification

The two grids compared are the final occupancy-probability maps of the matched multi-robot *dynamic baseline* simulation (section S5): the Bayesian arm and the belief-function arm fused under the pignistic-matched (BetP) sensor model, on the identical environment, trajectories, and LiDAR seed schedule. Using the matched maps is deliberate—it isolates the residual algorithmic difference rather than any sensor-model confound.

Each probability grid is converted to a binary obstacle map by a single occupancy threshold of 0.5: a cell is an obstacle iff its occupancy probability is ≥ 0.5. The robot is modeled as a point (no inflation radius), so the threshold is the only free parameter in the obstacle classification and it is identical for both arms.

S9.2 Start–Goal Pair Sampling

We sample $N = 500$ start–goal pairs with a fixed seed of 0 (NumPy `default_rng(0)`), making the pair set deterministic. To guarantee that neither arm is sampled into cells the other regards as occupied, candidate cells are drawn from the union obstacle map (a cell is admissible only if both arms classify it as free). Each pair is drawn by rejection sampling: two distinct free cells are proposed, and the pair is rejected and re-drawn if the separation between start and goal is below 5 cells, avoiding trivially short queries.

S9.3 Per-Pair Planner and Metrics

For each pair, $A^\star$ is run *independently* on each arm's binary obstacle map. The planner is 8-connected (cardinal cost 1, diagonal cost $\sqrt{2}$) with the admissible octile-distance heuristic. Clearance is computed once per arm as the exact Euclidean distance transform of the free space; the clearance of a path is its minimum cell-to-nearest-obstacle distance. The per-pair quantities, aggregated over the 500 pairs, are:

Shared reachability. A pair is jointly reachable iff $A^\star$ returns a valid path on *both* obstacle maps. The reported reachability is the fraction of pairs reachable by both arms.

Path equivalence. For jointly reachable pairs, the two paths are equivalent iff their symmetric Hausdorff distance is ≤ 1 cell. The path-equivalence rate is the fraction of jointly reachable pairs meeting this criterion.

Mean path-length difference. The mean over jointly reachable pairs of the absolute difference $|\ell_B - \ell_{\mathrm{DS}}|$ in path length (cells).

Clearance difference. The mean over jointly reachable pairs of the absolute difference $|c_B - c_{\mathrm{DS}}|$ between the two paths' minimum clearances (cells).

Safety-critical clearance disagreement. A pair is flagged safety-critical iff one arm finds a path and the other does not, *or* both arms find paths but their minimum clearances differ by more than 1 cell. The reported rate is the fraction of all 500 pairs so flagged.

S9.4 Results

Across the 500 start–goal pairs, shared reachability is 100% (500/500 pairs reachable by both arms; per-arm planning success 1.00). Path equivalence holds for 77% of pairs (0.774). The mean

path-length difference is 0.08 cells and the mean clearance difference is 0.36 cells. A safety-critical clearance disagreement occurs in 7% of pairs (0.07). The map-level differences therefore leave the navigation outcome almost entirely intact—both arms reach every goal, agree on the route in over three-quarters of queries, and produce paths whose lengths differ by a fraction of a cell on average—while the 7% safety-critical rate confirms that the residual boundary-sharpness difference has a measurable but small footprint near obstacles, not one that compromises reachability or path length.

References

George Casella and Roger L. Berger. *Statistical Inference*. Duxbury/Thomson Learning, Pacific Grove, CA, 2 edition, 2002.

Larry V Hedges and Ingram Olkin. *Statistical Methods for Meta-Analysis*. Academic Press, 1985.

Sture Holm. A simple sequentially rejective multiple test procedure. *Scandinavian Journal of Statistics*, 6(2):65–70, 1979.

Arthur Huletski, Dmitriy Kartashov, and Kirill Krinkin. VinySLAM: An indoor SLAM method for low-cost platforms based on the Transferable Belief Model. In *2017 IEEE/RSJ International Conference on Intelligent Robots and Systems (IROS)*, pages 6770–6776. IEEE, 2017. doi: 10.1109/IROS.2017.8206595.

Daniel Lakens. Equivalence tests: A practical primer for t tests, correlations, and meta-analyses. *Social Psychological and Personality Science*, 8(4):355–362, 2017. doi: 10.1177/1948550617697177.

Julien Moras, Véronique Cherfaoui, and Philippe Bonnifait. Credibilist occupancy grids for vehicle perception in dynamic environments. In *2011 IEEE International Conference on Robotics and Automation (ICRA)*, pages 84–89. IEEE, 2011a. doi: 10.1109/ICRA.2011.5980298.

Julien Moras, Véronique Cherfaoui, and Philippe Bonnifait. Moving objects detection by conflict analysis in evidential grids. In *2011 IEEE Intelligent Vehicles Symposium (IV)*, pages 1122–1127. IEEE, 2011b. doi: 10.1109/IVS.2011.5940561.

Dominik Nuss, Stephan Reuter, Markus Thom, Ting Yuan, Gunther Krehl, Michael Maile, Axel Gern, and Klaus Dietmayer. A random finite set approach for dynamic occupancy grid maps with real-time application. *The International Journal of Robotics Research*, 37(8):841–866, 2018. doi: 10.1177/0278364918775523.

Donald J Schuirmann. A comparison of the two one-sided tests procedure and the power approach for assessing the equivalence of average bioavailability. *Journal of Pharmacokinetics and Biopharmaceutics*, 15(6):657–680, 1987. doi: 10.1007/BF01068419.

James H Steiger. Beyond the F test: Effect size confidence intervals and tests of close fit in the analysis of variance and contrast analysis. *Psychological Methods*, 9(2):164–182, 2004. doi: 10.1037/1082-989X.9.2.164.

Ronald R Yager. On the Dempster-Shafer framework and new combination rules. *Information Sciences*, 41(2):93–137, 1987. doi: 10.1016/0020-0255(87)90007-7.
